# Ethanol-induced CYP2E1 Expression is Reduced by Lauric Acid via PI3K Pathway in HepG2 Cells

**DOI:** 10.21315/tlsr2020.31.3.5

**Published:** 2020-10-15

**Authors:** Ying-Huan Lua, Wei-Wah Ong, Hong-Kin Wong, Choy-Hoong Chew

**Affiliations:** Department of Allied Health Sciences, Faculty of Science, Universiti Tunku Abdul Rahman, Jalan Universiti, Bandar Barat, 31900 Kampar, Perak, Malaysia

**Keywords:** Fatty Liver, Alcoholic Liver Disease, Cytochrome P450, Medium Chain Fatty Acid, Antioxidant, Hati Berlemak, Penyakit Hati Berlemak Beralkohol, Cytochrome P450, *Medium Chain Fatty Acid*, Antioksidan

## Abstract

The metabolism of alcohol involves cytochrome P450 2E1 (CYP2E1)-induced oxidative stress, with the association of phosphatidylinositol-3-kinases (PI3K) and nuclear factor kappa B (NFκB) signalling pathways. CYP2E1 is primarily involved in the microsomal ethanol oxidising system, which generates massive reactive oxygen species (ROS) and ultimately leads to oxidative stress and tissue damage. Lauric acid, a major fatty acid in palm kernel oil, has been shown as a potential antioxidant. Here, we aimed to evaluate the use of lauric acid as a potential antioxidant against ethanol-mediated oxidative stress by investigating its effect on CYP2E1 mRNA expression and the signalling pathway in ethanol-induced HepG2 cells. HepG2 cells were firstly treated with different concentrations of ethanol, and subsequently co-treated with different concentrations of lauric acid for 24 h. Total cellular RNA and total protein were extracted, and qPCR and Western blot was carried out. Ethanol induced the mRNA expression of CYP2E1 significantly, but lauric acid was able to downregulate the induced CYP2E1 expression in a dose-dependent manner. Similarly, Western blot analysis and densitometry analysis showed that the phosphorylated PI3K p85 (Tyr458) protein was significantly elevated in ethanol-treated HepG2 cells, but co-treatment with lauric acid repressed the activation of PI3K. However, there was no significant difference in NFκB pathway, in which the normalised NFκB p105 (Ser933) phosphorylation remained constant in any treatment conditions in this study. This suggests that ethanol induced CYP2E1 expression by activating PI3K p85 (Tyr458) pathway, but not the NFκB p105 (Ser933) pathway in HepG2 cells.

## Abstract (ms)

Metabolisme alkohol melibatkan tekanan oksidatif akibat cytochrome P450 2E1 (CYP2E1), dengan penglibatan phosphatidylinositol-3-kinases (PI3K) and faktor nuklear kappa B (NFκB). CYP2E1 memainkan peranan penting dalam sistem pengoksidaan etanol microsomal, yang akan menghasilkan spesies oksigen reaktif (ROS) dan akhirnya membawa kepada kerosakan tisu. Asid laurik, yang merupakan asid lemak utama dalam minyak isirong sawit, telah terbukti memperoleh potensi antioksidan. Kajian ini bertujuan untuk menilai penggunaan asid laurik sebagai antioksidan terhadap tekanan oksidatif yang dirangsangkan oleh etanol. Kami menyiasat kesan asid laurik terhadap ekspresi mRNA CYP2E1 dan laluan isyarat yang disebabkan oleh etanol dalam sel HepG2. Justeru, HepG2 dirawat dengan kepekatan etanol yang berbeza, dan seterusnya dirawat dengan kepekatan asid laurik yang berbeza selama 24 jam. Jumlah RNA dan jumlah protein telah diekstrak, dan qPCR dan blot western telah dijalankan. Etanol mendorong ekspresi mRNA CYP2E1 dengan ketara, tetapi asid laurik dapat mengimbangi ekspresi CYP2E1. Analisis blot western dan analisis densitometri menunjukkan bahawa tahap protein fosforilasi PI3K p85 (Tyr458) telah meningkat dengan ketara dalam sel-sel HepG2 yang dirawat etanol, tetapi rawatan bersama dengan asid laurik menindas pengaktifannya. Walau bagaimanapun, laluan NFκB tidak menunjukkan sebarang perbezaan. Tahap fosforilasi NFκB p105 (Ser933) kekal sama dengan sebarang keadaan rawatan dalam kajian ini. Ini menunjukkan bahawa etanol merangsangkan expresi CYP2E1 dengan mengaktifkan laluan PI3K p85 (Tyr458), tetapi bukan melalui NFκB p105 (Ser933) dalam sel HepG2. Asid laurik menindas rangsangan etanol dengan menghentikan pengaktifan laluan PI3K.

**Highlights**

- Lauric acid downregulated ethanol-induced CYP2E1 expression in a dose-dependent manner.
- Ethanol induced CYP2E1 expression by activating PI3K p85 (Tyr458) pathway in HepG2 cells.
- Co-treatment with lauric acid repressed the activation of PI3K, but not the NFκB pathway.

## INTRODUCTION

The harmful consumption of alcohol has resulted in three million deaths worldwide and 132.6 million disability-adjusted life years in 2016 (World Health Organisation 2018). Despite the beneficial roles of moderate alcohol consumption, excessive alcohol intake is detrimental to health and it can lead to various health problems especially alcoholic liver disease (ALD). The spectrum of liver diseases encompassed by ALD are steatosis, hepatitis and cirrhosis.

Alcohol metabolism involves cytochrome P450 2E1 (CYP2E1) enzyme and this enzyme generates acetaldehyde as well as reactive oxygen species (ROS) which lead to ALD (Koop 2006). Numerous studies have proven the increment of CYP2E1 mRNA and protein expressions upon ethanol exposure (Lieber 1997; Jin *et al*. 2013; Bak *et al*. 2016). CYP2E1 accounts for 10% of total alcohol metabolism under normal circumstances. CYP2E1-mediated alcohol metabolism generates massive amount of reactive oxygen species (ROS) and acetaldehyde which increase the extent of liver injury and lead to the progression of ALD (Koop 2006). Ethanol administration was shown to increase CYP2E1 in wild-type mouse by five-fold but not in CYP2E1 knockout mouse (Bardag-Gorce *et al*. 2000). In CYP2E1 knock-in mice, ethanol administration led to the accumulation of lipid and steatosis as compared to CYP2E1 knockout mice (Wu *et al*. 2012). The rise in CYP2E1 expression is hypothesised to be associated with activation of phosphatidylinositol-3-kinases (PI3K) and nuclear factor kappa B (NFκB) pathways as these pathways are involved in alcohol induction (Mandrekar & Szabo 2009; Zeng *et al*. 2012; Wang *et al*. 2015; Zeng *et al*. 2018).

Current treatment and prevention of ALD involves the application of antioxidants to protect liver from alcohol-mediated oxidative damage. Well studied antioxidants from plant sources such as quercetin and resveratrol, are now in clinical trials for anti-oxidative therapy in liver disease (Li *et al*. 2015). Our study focuses on lauric acid, a 12-carbon saturated medium chain fatty acid (MCFA). Lauric acid is the main saturated fatty acid that found abundantly in coconut oil and palm oil (Fife 2013). Although lauric acid is a saturated fatty acid, it is less obesogenic as compared to long chain fatty acid (LCFA) intakes (McCarty & DiNicolantonio 2016). Saturated fats have been shown to alleviate cardiovascular diseases, obesity as well as hypercholesterolemia (Siri-Tarino *et al*. 2010). Lauric acid was shown to reduce the production of superoxide in spontaneously hypertensive rats (Alves *et al*. 2015). Experiments using virgin coconut oil, a product which is rich in lauric acid, also reduced the oxidative stress, increased catalase and superoxide dismutase (SOD) activities (Alves *et al*. 2015; Nevin & Rajamohan 2006; Famurewa *et al*. 2018). Recent studies have also demonstrated anti-inflammatory, anti-oxidative and anti-atherosclerotic activities of lauric acid (Cheah *et al*. 2014; Lim *et al*. 2015; Ong *et al*. 2019). Here we show that lauric acid abolished ethanol-induction of CYP2E1 through PI3K pathway. Understanding on the underlying signalling pathways utilised by lauric acid would definitely contribute to its future role as a therapeutic agent for treating the effects of alcoholism.

## METHODS

### Cell Culture and Treatment

HepG2 cells were obtained from American Type Culture Collection (ATCC) and grown as previously reported (Lim *et al*. 2013). A number of 2.0 × 10^6^ of cells was seeded into each 25 cm^2^ tissue culture flasks and allowed to grow to 70% confluence. The cells in different flasks were then treated with different concentrations [1% (v/v), 2% (v/v), 5% (v/v) and 10% (v/v)] of ethanol (EMPARTA^®^, Germany). The control cells were cultured with fresh medium without the addition of ethanol. These culture flasks were incubated in 5% CO_2_ incubator at 37°C for 24 h. For subsequent experiments, the cells were treated with 2% (v/v) ethanol in addition to different concentrations (5 μM, 10 μM and 20 μM) of lauric acid (Sigma Aldrich, USA). For positive control, 20 μM of resveratrol was used and co-treated with ethanol, while in the vehicle control, the diluent for lauric acid was used.

### RNA Extraction

After 24 h of treatment, total cellular RNA was extracted from HepG2 cells using Tri-Reagent^®^ LS (Molecular Research Center, USA). Electrophoresis of RNA was carried out by using 1% (w/v) denaturing agarose-bleach gel to check the integrity of isolated RNA. The concentration of isolated total cellular RNA was measured using NanoPhotometer (Implen, Germany). RNA purity was also checked by measuring optical density at 260 nm and 280 nm. DNase treatment was carried out by using RQ1 RNase-Free DNase (Promega, USA).

### qRT-PCR

qPCR was performed by using KOD SYBR^®^ Green Master Mix (Toyobo, Japan) in order to quantify the CYP2E1 mRNA expression after treatment using CFX96™ Real-Time PCR Detection System (BioRad, USA). Primer sequences used for CYP2E1 are: 5′-AATGGACCTACCTGGAAGGAC-3′ and 5′-CCTCTGGATCCGGCTCTCATT-3′. GAPDH was used as housekeeping gene and the primer sequences are: 5′-GAAGGTGAAGGTCGGAGTC-3′ and 5′-GAAGATGGTGATGGGATTTC-3′. The following parameters was used for the PCR: 20 min at 61°C, 30 s at 95°C, followed by 40 cycles of 30 s at 94°C, 30 s at 57°C, and 45 s at 72°C, before melt curve analysis was performed.

### Protein Isolation and Quantification

Protein was extracted using TRI-Reagent^®^ LS following manufacturer’s instructions. The concentration of extracted protein was determined using Bio-Rad’s DC Protein Assay reagent kit (Lim *et al*. 2013; Ong *et al*. 2019).

### Western Blot

After SDS-PAGE electrophoresis, wet transfer was carried out to transfer the proteins from the gel to a polyvinylidene fluoride (PVDF) (Millipore, USA) membrane using Mini Trans-Blot^®^ Electrophoretic Transfer Cell (BioRad, USA). The primary antibodies used were NFκB p105/50 antibody, phospho-NFκB p105 (Ser933) antibody, PI3K p85 antibody, phospho-PI3K p85 (Tyr458)/p55 (Tyr199) antibody, and β-actin antibody (Cell Signaling, USA). All of the primary antibodies were diluted in 1:1000 except β-actin antibody which was diluted in a ratio of 1:1500. Chemiluminescence detection was done using ChemiDoc^TM^ XRS+ System (Bio-Rad, USA) and Immobilon Western Chemiluminescent HRP substrate (Millipore, USA). Image Lab^TM^ version 6.0 software (Bio-Rad, USA) was used for the quantification of the immunodetected protein bands. The expression of each protein was quantified and normalised against the protein expressions of their respective total protein and housekeeping gene, β-actin.

### Statistical Analysis

Statistical Package for the Social Sciences (SPSS) version 22 software was used in this study to calculate the independent-samples *t*-test. Statistically significant result was indicated with *p* < 0.05 while *p* < 0.01 indicated statistically very significant.

## RESULTS

### CYP2E1 is Induced by Ethanol

**Figure 1:**
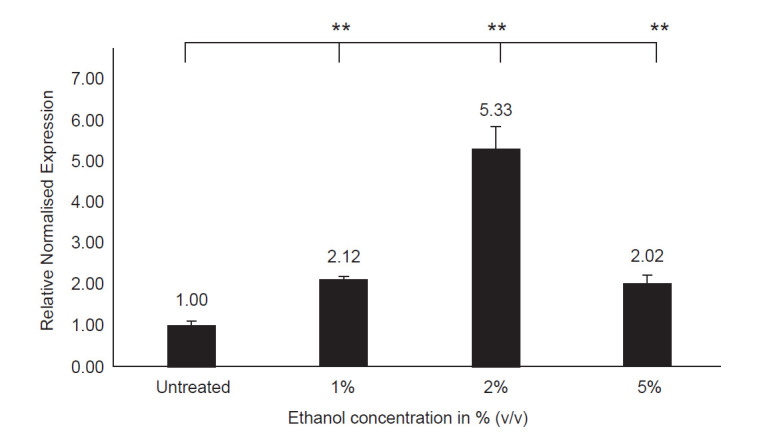
Graphical representation showing CYP2E1 expression in HepG2 cells treated with 1% (v/v), 2% (v/v) and 5% (v/v) of ethanol (*n* = 2). The value above each bar shows the fold-change of normalised CYP2E1 expression in relative to vehicle control (1.00-fold). The error bars represent the standard deviation with the values expressed as mean SD; **p-value < 0.01 indicates statistical significance.

CYP2E1 mRNA expression was found to be induced by alcohol (Fig. 1). In 1% (v/v), 2% (v/v) and 5% (v/v) ethanol-treated cells, the CYP2E1 mRNA expression was increased to 2.12-fold, 5.33-fold and 2.02-fold, respectively. The results are in concordance to the previous reported studies (Raucy *et al*. 2004). Surprisingly, CYP2E1 mRNA expression was decreased in 5% (v/v) of ethanol and this could be due to the toxicity from high concentration of ethanol that would decrease the number of viable cells. This was later confirmed using cell viability test (Ong *et al*. 2019). Therefore, the optimal concentration of alcohol induction in HepG2 cells was fixed at 2% (v/v) for all subsequent experiments.

### Lauric Acid Abolishes Ethanol-induced CYP2E1 mRNA Expression

**Figure 2:**
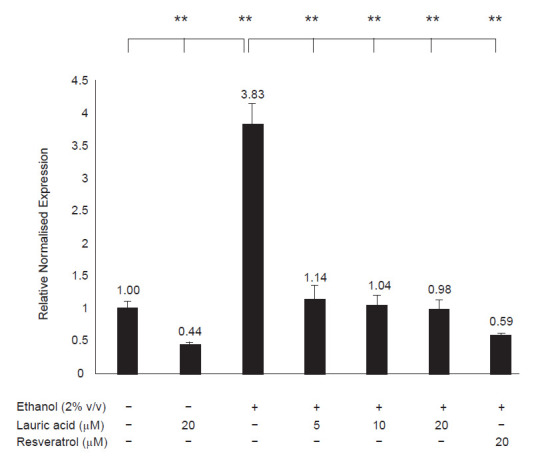
Graphical representation showing CYP2E1 expression in HepG2 cells treated with/without 2% (v/v) ethanol, 5 μM, 10 μM and 20 μM of lauric acid, and 20 μM of resveratrol (*n* = 2). The value above each bar shows the fold-change of CYP2E1 expression which was normalised to GAPDH and relative to vehicle control (1.00-fold). The error bars represent the standard deviation with the values expressed as mean SD; **p < 0.01 indicates statistical significance.

In the presence of lauric acid, the ethanol-induced CYP2E1 mRNA expression was downregulated in a dose-response manner. Fig. 2 shows that the CYP2E1 mRNA expression was reduced to basal level when increasing dose of lauric acid was added to the cells (1.14-fold, 1.04-fold and 0.98-fold). Resveratrol on the other hand reduced ethanol-induced CYP2E1 mRNA expression to almost half of the basal expression (0.59-fold).

### Ethanol Induces CYP2E1 Expression via PI3K Pathway but Lauric Acid Blocks the Activation

**Figure 3:**
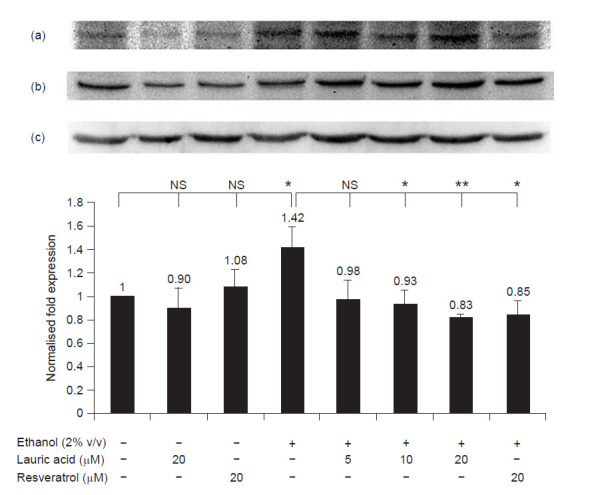
Chemiluminescent detection and graphical representation of the quantitative protein expression of phosphorylated PI3K p85 (Tyr458) in HepG2 cells (*n* = 3). (a) Phosphorylated PI3K p85 (Tyr458), (b) PI3K p85 and (c) β actin protein bands. Error bars are expressed as standard deviation and the data represent mean ± SD; *n* = 3. *p < 0.05 and **p < 0.01 represent the statistically significant changes in the expression. NS represents non-significance.

**Figure 4:**
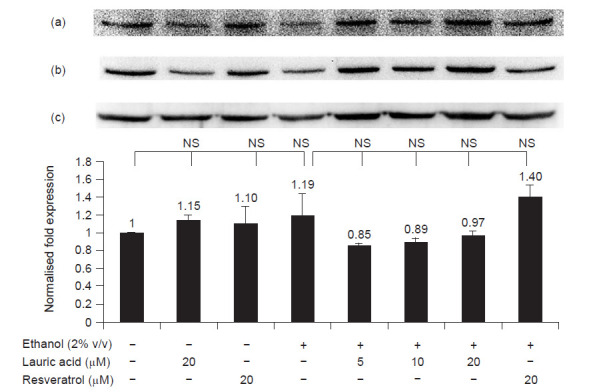
Chemiluminescent detection and graphical representation of the quantitative protein expression of phosphorylated NFκB p105 (Ser933) in HepG2 cells (*n* = 3). (a) Phosphorylated NFκB p105 (Ser933), (b) NFκB p105 and (c) β actin protein bands. Error bars are expressed as standard deviation and the data represent mean ± SD; n = 3. NS represents non-significance.

Here, we also show that ethanol induced CYP2E1 expression via PI3K pathway and lauric acid attenuated the induction by abolishing the activation of PI3K pathway (Fig. 3). Lauric acid inactivated the pathway by blocking the expression of phosphorylated PI3K p85 (Tyr458) in a dose dependent manner. However, there were no changes observed in NFκB pathway (Fig. 4). On the other hand, resveratrol produced contradicting results on both pathways. While it had no effect on NFκB pathway (Fig. 4), resveratrol acted similarly to lauric acid by abolishing the ethanol activation of PI3K pathway (Fig. 3).

## DISCUSSION

A few studies have reported the induction of CYP2E1 mRNA by fatty acids such as palmitic acid (Raucy *et al*. 2004) and oleic acid (Mahli *et al*. 2015). Besides that, oleic acid was shown to work synergistically with alcohol in inducing the CYP2E1 mRNA expression (Mahli *et al*. 2015). Interestingly, we show here that lauric acid demonstrated a complete opposite effect to both of the fatty acids. Instead, lauric acid comes with antagonistic effect to the action of alcohol by significantly suppressing the expression of alcohol-induced CYP2E1 mRNA in this current study.

Recent studies have discovered signalling pathways which are involved in the regulation of alcohol-mediated CYP2E1 such as NFκB and PKC/JNK/SP1 pathway (Jin *et al*. 2013; Lin *et al*. 2019). NF-κB serves as the transcription factor of CYP2E1 and apolipoprotein A-I (Zordoky & El-Kadi 2009; Tong *et al*. 2019) and is induced by alcohol, which further activates its downstream cyclooxygenase-1 (COX-1) and inducible nitric oxide synthase (iNOS) expression (Blanco *et al*. 2005). The activation of NF-κB is guarded by its inhibitor, IκBα. When IκBα interacts with p65, NF-κB will be held as inactive. Phosphorylation of IκBα will result in its degradation of NF-κB and abolish the suppression effect (Mahli *et al*. 2015). Furthermore, low CYP2E1 gene expression has been associated with an increased autophagy and hepatic nuclear factor E2-related factor 2 (Nrf2) expression, mediated by PI3K/Akt-p38 MAPK (Cederbaum 2013; Bak *et al*. 2016). In alcoholic cardiomyopathy, apoptosis and necrosis are attributed to autophagy (Piano & Philips 2014), although the molecular mechanisms and signalling pathway involved remain elusive (Fernández-Solà 2020).

This result was in agreement with previous study which showed PI3K signalling pathway mediates the effect of alcohol (Umoh *et al*. 2014; Laguesse *et al*. 2017). Alcohol has been shown to specifically activated the PI3K/AKT pathway in this brain region of mice (Neasta *et al*. 2011). Interestingly, recent works also showed that the need for alcohol consumption could be reduced by inhibiting fibroblast growth factor 2 (FGF2) expression via PI3K signalling (Even-Chen & Barak 2019). Early works have demonstrated that activation of PI3K pathway happens via phosphorylation of tyrosine residues in the SH2 domain of p85 (Yu *et al*. 1998). The tyrosine residue identified from this study was at the position 458. Likewise, the activation of this pathway might be due to the improved stoichiometry between p110 and p85 subunits when the PI3K p85 subunit is reduced (Zeng *et al*. 2012), which was correlated with this study. Activation of PI3K pathway might due to formation of ROS as a consequence of alcoholism (Zhang *et al*. 2016). During alcohol metabolism, acetaldehyde has been shown to increase the amount of mature sterol regulatory element-binding protein (SREBP) by decreasing the sterol concentration (You *et al*. 2002; Wada *et al*. 2008). The regulation of SREBP activity in hepatic lipogenesis which may contribute to alcoholic fatty liver development is controlled by PI3K/Akt pathway (Porstmann *et al*. 2008; Xia *et al*. 2020). Hence, PI3K pathway might be activated in this study in order to regulate SREBP in response to ethanol exposure.

Nevertheless, there was no statistically significant changes by any of the treatments on NFκB pathway (Fig. 4). The insignificant results obtained here is in discrepancy with previous studies which proved that chronic ethanol induced accumulation of ROS and activated NFκB signalling pathway to trigger inflammation (Wang *et al*. 2015). Acute ethanol exposure activates NFκB in astrocytes (Blanco *et al*. 2005). However, acute ethanol treatment has been found to reduce the NFκB phosphorylation at the site of NFκB p65 Ser536 in human monocytes instead (Mandrekar *et al*. 2007). Thus, NFκB pathway could be cell line specific which could explain the non-significant effect observed in HepG2 cells in this study.

Acute alcoholism causes the elevation of CYP2E1, oxidative stress and lipogenic SREBP, which consequently lead to steatosis (Purohit *et al*. 2008). As mentioned before, SREBP-1 activity can be enhanced via PI3K/Akt signalling pathway. Thus, the antioxidant property of lauric acid may possibly reduce the activity of SREBP through suppression of PI3K signalling pathway. Suppression of PI3K pathway would eventually reduce the activity of mammalian target of rapamycin (mTOR) and signal transducer and activator of transcription 3 (STAT3), and the subsequent transcription of CYP2E1 (Patel *et al*. 2014). Although rat CYP2E1 transcription regulation is shown to be mediated by NFκB pathway (Lin *et al*. 2019), the results from this study shows that involvement of this pathway is not prominent in HepG2 cells.

## CONCLUSION

Here, we proved that lauric acid abolished the phosphorylation of PI3K p85 at Tyr458 by ethanol, and this is the most likely mechanism behind the reduction of CYP2E1 expression in HepG2 cells.
